# Risk characterization for silica-related silicosis and lung cancer in communities adjacent to sand and gravel extraction facilities: examining limitations in our current risk methods

**DOI:** 10.3389/fpubh.2025.1558778

**Published:** 2025-06-18

**Authors:** Anthony J. Russell, Kenneth A. Mundt, Andrew Maier

**Affiliations:** ^1^Integral Consulting, Los Angeles, CA, United States; ^2^Department of Biostatistics and Epidemiology, School of Public Health and Health Sciences, University of Massachusetts, Amherst, MA, United States; ^3^Integral Consulting, Cincinnati, OH, United States

**Keywords:** silica, silicosis, lung cancer, risk assessment, risk characterization, community exposure

## Abstract

**Introduction:**

Effectively managing and reducing occupational exposure to crystalline silica continues to be a critical priority for public health agencies. The relationship between workplace inhalation exposure to respirable crystalline silica (RCS) and the onset of silicosis is well known. The body of evidence has shaped the evolution and promulgation of specific standards for the assessment and control of workplace RCS exposures. However, ongoing health effects research continues to explore the impacts of the physical and chemical forms of crystalline silica and the potential of RCS to cause lung-disease. Further, the levels at which occupational RCS exposure potentially increases the risk of lung cancer in occupational settings remains uncertain. Even less is known of the risk of lung disease associated with community level exposure to RCS. This investigation examines the implications for assessing community exposure and silicosis and lung cancer risk from: (1) direct quantitative application of occupational epidemiology data using non-threshold assumptions, and (2) application of threshold-like risk assessment approaches informed by mode of action hypotheses. An evidence integration approach is proposed with refinements to traditional methods that incorporates updates for scenario extrapolation based on the hazard quotient (HQ) approach. The approach extends the traditional application of adjustment factors in extrapolating from occupational dose–response data to include three modifying factors that address scenario-relevant data on inhalation dosimetry, exposure intensity, and relative potency of RCS forms.

**Methods:**

Community RCS exposures adjacent to National Sand, Stone, and Gravel Association (NSSGA) member facilities were estimated from exposure levels a forthcoming publication. The exposure levels were supplemented by publicly available data from state and federal governing bodies. Three modifying factors were hypothesized as key in determining the disease-causing potential of RCS from community exposures when extrapolating from occupational epidemiology findings. The analysis and review of the literature focused on the current research outlining each of the three factors. RCS dose-response data for lung cancer and silicosis were obtained from a recent systematic review and supplemented by web-based literature searches. Traditional risk assessment methods were applied to the epidemiological study results. For cancer risk based on linear dose–response assumptions, theoretical risk estimates for ambient and background levels of RCS were calculated using an occupational epidemiology-based regression equation. Further, traditional health-based limits derived from a point of departure and application of adjustment factors were also used to assess alignment of traditional risk assessment tools and observations regarding community risks.

**Results:**

Occupational epidemiological studies included in the analysis were mixed, some showing a significant positive relationship between increasing silica exposure and lung cancer risk. However, other reviews have found no dose–response relationship between silica and lung cancer. *In-vivo* dose–response studies in animals of RCS and lung cancer were similarly limited. All studies showed increased silicosis risks above various estimated thresholds. To explore the potential difference between silicosis and lung cancer risks at the general population level, a linear regression for lung cancer risk and cumulative exposure to RCS was performed. The regression analysis resulted in a lung cancer risk = 0.069(mg/m^3^) X + 1.17 (*p* = 0.017) and a silicosis risk = 1.75 (mg/m^3^) X + 1.67 (*p* < 0.001). At the community background exposure equivalent of 4 μg/m^3^, these regression analyses resulted in a RR for lung cancer of 1.17 (95% CI: 1.169–1.171) and a RR for silicosis of 1.68 (95% CI: 1.66–1.69). Mode of action analyses supported a threshold-like response for lung cancer with inflammatory markers being key drivers. The upper end of the range of HQs derived by comparing the range of community exposures to health-based limits for lung effects of RCS exceeded the target value of unity. These, estimates do not align with the lack of observed effects in communities adjacent to sand, stone, and gravel operations or locations with high background levels of ambient RCS.

**Conclusion:**

Risk of silicosis and lung cancer due to community exposures to RCS need to be addressed in the context of observable increases of disease in populations exposed to low to moderate levels of RCS. Direct epidemiology studies of community exposures are lacking, but no clear indication of concern based on lung effect prevalence has been reported. In the absence of such data, extrapolations for risk assessment using occupational epidemiology data as a basis for potency estimates are used and need further adjustments. Application of such adjustments would likely support a conclusion that community exposures do not exceed the threshold necessary for carcinogenesis to observe elevated levels of lung cancer or for silicosis. This analysis supports a modified HQ or modified Margin of Safety (MOS) approach with additional modifiers including inhalation dosimetry, exposure intensity and potency that address differences between occupational and community exposure scenarios.

## 1 Introduction

Effectively managing and reducing occupational exposure to crystalline silica continues to be a critical priority that has been the subject of public health science and media attention.^1^^,^^2^ The exposure profiles among various industry sectors, toxicologic potency and mode of action data, epidemiology study findings, and risk characterizations have been published in numerous comprehensive reviews by government agencies and in the peer-reviewed literature over the last 10 years (1–4). The relationship between workplace inhalation exposure to crystalline silica in the respirable size fraction (RCS) and the onset of silicosis has been known historically and studied systematically for decades with increasing risk management activity. It is also known that the intensity of exposure and cumulative magnitude of exposure show a threshold dose–response relationship with silicosis incidence and severity (5, 6). The body of evidence has shaped the evolution and promulgation of specific standards for the assessment and control of workplace RCS exposures. In the U.S. this was most recently reflected in the promulgation of an updated workplace silica standard, reducing the permissible exposure limit (PEL) from an equivalent of 100 μg/m^3^ in general industry and 250 μg/m^3^ in construction, to 50 μg/m^3^ RCS with an action level of 25 μg/m^3^ in all industries (4). Despite this progress, some researchers argue that current occupational exposure limits may still be insufficient to protect workers’ health and that implementing stricter exposure limits could potentially reduce silica-associated mortality (7–9).

While much is known about the occupational risks for silicosis and other non-cancer systemic effects associated with occupational exposures to RCS, there are many current uncertainties. Ongoing health effects research continues to explore the impacts of physical and chemical forms of crystalline silica on RCS potency (10–13), the underlying toxicological mode of action driving these effects (12, 14–18), and the presence and best estimate for a threshold concentration (and associated deposited dose) for onset of non-malignant diseases (19, 20), among other considerations. At what occupational levels increase lung cancer risk is increased (if at all) remains uncertain.

The evidence for a relationship between occupational exposure to RCS and increased lung cancer risk is mixed. Further dose–response results for RCS and lung cancer remains uncertain. Numerous agencies have reviewed the risk profile of silica and lung cancer but to date have not established a carcinogenic dose response curve (1, 2, 21, 83). Indeed, regarding the exposure-response relationship between crystalline silica and lung cancer ATSDR states “additional well-controlled occupational exposure studies would provide important information regarding exposure-response relationship for c-silica-induced lung cancer and the relationship between silicosis and lung cancer” (1). IARC (2) and NTP (83) do not provide a dose–response approach or potency estimate as these agencies historically provided only hazard classifications and characterizations. OSHA (4) reported an estimated threshold effect for inflammation and carcinogenesis of 36 μg/m^3^ over a 45-year working lifetime. None of these regulatory agencies provide a dose–response relationship for RCS induced lung cancer.

From a mode of action (MOA) view, potential relationships predicated on arguments related to secondary impacts of inflammation, cytotoxicity, and oxidative stress are appealing from a biological plausibility perspective (14–16, 18, 22, 23). Further, animal bioassays, while limited, may support the carcinogenicity of RCS in rats, but not in mice or hamsters (2, 24–28). Among these studies, 7 were full lifetime cancer bioassays (recovery periods varied but were either 6 weeks, 4, 8, 12, 16, or 34 months after exposure). In these studies, increase in lung tumors was found among rats (but not hamsters or mice). Further, exposure to RCS in the occupational epidemiology literature provides mixed results with some studies indicating a potential increased risk, particularly among workers with silicosis (29, 30). Further, some investigators have concluded that exposure to silica at levels above the current occupational exposure limits can lead to silicosis, lung cancer, and other respiratory diseases (8, 31). However, other well-designed studies have not shown such an association between RCS exposure and cancer, even for exposure groups that do have an increased silicosis risk (14, 19, 32–38). Thus, while plausible MOA hypotheses have been suggested, the *in vivo* toxicology and epidemiology provide less-compelling results.

Further complicating the mixed results in occupational epidemiology, is the translation of such data to the general population or community level exposure scenarios. This manuscript examines the relevance and applicability of the relationships reported between historical occupational RCS exposure and silicosis and lung cancer risks to the significantly lower RCS exposure concentrations measured in communities surrounding and at the fence-line of sand, stone, and gravel extraction facilities. Reliance on occupational epidemiology data is necessary, in the absence of community epidemiological studies having sufficient statistical power to address potentially increased cancer risks [(2), pp. 367–358]. Specifically, this analysis examines the implications for community exposure cancer risk assessment from: (1) direct quantitative application of occupational epidemiology data, and (2) application of threshold-like risk assessment approaches informed by mode of action hypotheses. The application of these traditional and simplistic extrapolations can generate misalignments between the predicted community risk and what is observed. This analysis proposes an evidence integration approach with refinements to traditional methods that incorporates modifiers for scenario extrapolation based on RCS inhalation dosimetry, exposure intensity, and relative potency.

The goal of addressing community risks reflects growing interest in understanding the impacts of commercial sand, stone, and gravel operations on community health.^3^^,^^4^^,^^5^^,^^6^ Numerous studies have published RCS exposure data for community background as well as adjacent to commercial facilities (1, 39). Thus, contextualizing such exposure data is an important public health activity. Providing clear and transparent risk analyses supports communication and engagement among commercial and community partnerships and assessment on resource allocation to support community health and well-being. Therefore, our goal was to conduct a global view of potential risks across diverse sites and not to conduct an updated site-specific *de novo* risk assessment. Rather we use the established linear-no-threshold approach for cancer risk assessment as well as existing community exposure limits based on point of departure and adjustment factor methods and compare the results to real world exposure data (39).

## 2 Methods

### 2.1 RCS exposure levels

For the current analysis, community RCS exposures adjacent to National Sand, Stone, and Gravel Association (NSSGA) member facilities were estimated from exposure levels reported in Richards and Brozell (39). The exposure levels were supplemented by publicly available data from state and federal governing bodies such as EPA, OSHA, TCEQ, CalEPA, and state agency community reviews (Table 1). Web-based literature searches were conducted for reports and publications that characterized background ambient levels of RCS. Search strings included “silica,” “quartz,” “silica dust” AND “ambient,” “background,” “general population.” Abstracts and titles were screened, and relevant publications were reviewed in depth for reported RCS levels.

**Table 1:** Summary adapted from Richards and Brozell (39) (Table 3-1).

Average quartz concentrations in ambient air in 22 US cities			
States	Avg. # of samples	Avg. coarse quartz ug/m^3^ (>PM 2.5 - < PM15)	Avg. fine quartz ug/m^3^ (PM2.5)
22 States from Boston, MA to five points, CA	5	3.5	0.36
Range		1.0–8.0	0.0–1.9

### 2.2 Hypothesized factors influencing the disease-causing potential of RCS

We hypothesized that the following three factors may be key in determining the disease-causing potential of RCS and thus would need incorporated in extrapolations of occupational data to community health risk assessments. These factors were selected based on previous toxicological, industrial hygiene, and mineralogical knowledge and confirmed as potential modifying factors following focused literature review. Our analysis and review of the literature focused on analyzing the feasibility of and current research outlining each of the following three factors.

#### 2.2.1 Inhalation dosimetry

Differences in breathing rate influence accumulation of fine particles within the lung due to lung dosimetry; breathing rate during exposure may drive increased risk in occupational study groups. Additionally, particle size differs between occupational and ambient scenarios which may impact RCS toxicity (40). Inhalation dosimetry tools can be used to adjust for differences in worker versus community deposited dose of RCS. Particle size and particle distribution contribute significantly to the estimate of biological relevant deposited dose. While occupational and ambient RCS exposure can contribute to respiratory diseases from a biological plausibility perspective, particle size itself has been shown to be a crucial factor in disease outcomes (40). For example, nano and micro-particles have been shown to exhibit distinct cellular toxicity regardless of molecular chemistry (11). Therefore, adequately accounting for the physical characteristics of RCS in airborne dust is imperative on setting community health-based limits and better understanding the disease-causing potential of RCS.

#### 2.2.2 Intensity

It is known that silica exposure can occur in both occupational and ambient settings, with occupational exposure typically being higher and more strongly associated with silicosis (41, 42). Additionally, the intensity of RCS exposures influences the rapidity and frequency of silicosis (1). Accelerated silicosis is associated with intense short-term exposure to fine RCS particles and is a rapidly progressive form of silicosis (1). In general, rapid silicosis develops between 5 to 10 years after initial exposure, however it is typically associated with more moderate exposures than silicosis, potentially due to differences in the nature of exposure (e.g., intensity and particle size) (1). Ambient silica levels are lower, with current data suggesting that maintaining PM10 standards is adequate for protection against fibrotic effects in non-compromised individuals (42). However, of particular concern to evaluate is whether and the degree to which ambient silica levels exceed general background levels downwind of industrial sources (39, 43). Therefore, both occupational and ambient silica exposure may contribute to respiratory diseases if exposure levels are sufficient, with particle size being a crucial factor in determining health impacts (40). Further, the dose rate or intensity of exposures are likely quite different when comparing occupational and community exposures – and data comparing these differing scenarios are lacking.

#### 2.2.3 Potency

Potency of RCS is a function of particle size, shape, and mineralogical composition. Potency of exposure for a given RCS dose may influence lung effects as background populations are exposed to larger weathered particles while occupational populations are exposed to freshly fractured fine particles, (i.e., particles that may be more potent toxicants to the lung). The fibrogenic potential of crystalline silica can differ based on exposure circumstances (44). Weathering processes can alter the mineralogical composition of silica-containing materials, with quartz being replaced by opaline silica in some cases (45). Specifically, cristobalite and quartz are the most cytotoxic forms when compared to coesite, stishovite and tridymite (44). Quartz is the most common polymorph encountered in occupational settings and therefore the polymorph of most concern. However, it can be expected that ambient RCS exposure levels may not be polymorph specific, and it is unclear as to the exact polymorphic breakdown to which the general population is exposed. Current estimates indicate that background levels of silica contain quartz, coesite, stishovite and tridymite in various concentrations (44). Occupational cohorts may be exposed to a more potent compound on a mass-to-mass basis than the general community, however specific data on potency factors is lacking. The chemical reactivity and toxic properties of crystalline silica may be due to the chemical structure of RCS which is characterized by silonal groups (SiOH) which protrude from its crystal surface. When compared to the other forms of RCS, cristobalite and quartz have a higher density of silanol groups. However, the current weight of evidence from experimental studies *in vitro* and *in vivo* suggests no clear differences between the cytotoxic, inflammatory or fibrogenic properties between cristobalite and quartz (44). Thus, in assessing the relative potency of occupational versus community exposures the prevalence of different polymorphs and effects of aging on aerosol potency need to be considered.

### 2.3 Dose–response analyses

RCS dose–response data for lung cancer and silicosis were obtained from epidemiological studies reviewed in Mundt et al. (35). Tables 2, 3 report cumulative silica exposure levels (mg/m^3^ – years) and effect estimates from eight studies reporting silicosis among occupational populations and ten studies of lung cancer among occupational populations exposed to RCS, respectively. The works presented in Mundt et al. (35), typically were by age, occupation, job title, tobacco use and concomitant chemical exposures. Therefore, our goal was not to conduct a new site-specific risk assessment but rather to conduct a global view of potential risks across diverse sites. We relied on real-world exposure measurements from Richards and Brozell (39) and the cumulative silica exposure effect estimates from Mundt et al. (35). RCS cumulative exposure levels in mg/m^3^-yrs and silicosis and cancer were extracted from studies detailed in Tables 2, 3 respectively. We adjusted for exposure duration and standardized exposures to a cumulative exposure metric (mg/m^3^-years). Exposure levels and risks were then collated for dose–response analysis. Additional dose-response data were supplemented with web-based literature searches. PubMed was the primary search engine and search strings included the terms “silica,” “quartz” “silica dust” “respirable crystalline silica” “RCS” AND “lung,” “lung cancer” “carcinoma” and “carcinogen.” Data were analyzed using Rstudio (Version 4.4.2, Rstudio Team (85), Rstudio: Integrated Development for R. Rstudio, PBC). Linear regression analysis and plots were performed in Rstudio using ggplot2() and the lm() functions. Exposure data and relative risks were also analyzed via non-linear regression under a Poisson distribution using the glm() function in ggplot2 (available upon request). These results did not substantially improve the model fit (data not shown) and therefore the linear regression results are presented (see Table 4).

**Table 2:** Lifetime silicosis and lung cancer risk (4).

Silicosis lifetime risk (4)		Lung cancer lifetime risk (68)	
Exposure (45 yrs)	Risk	Smoking status	Risk
0.05 mg/m^3^	20–170 in 1,000	Male never smoker	13 in 1,000
0.1 mg/m^3^	60–773 in 1,000	Male current smoker	172 in 1,000

**Table 3:** Occupational and community health-based RCS exposure limits.

	Agency	Level (ug/m^3^)	Duration	Particle size	Basis	Cancer affects (Y/N)
Community ambient air levels	CalEPA	3	Chronic	PM4	N/A	N
	Idaho DEQ	2.5 cristobalite, tridymite 5 (quartz, tripoli)	24-h	NA	N/A	N
	Indiana IDEM	3.1 (indoor)	Chronic	PM4	Adopted CalEPA	N
	Maryland MDE	0.25	Chronic	PM4	Derived from ACGIH TLV-TWA of 25 ug/m3	N
	Michigan DEGLE	3	Chronic	PM4	Adopted CalEPA	N
	Minnesota Pollution Control Agency	3	Chronic	PM4	Adopted CalEPA	N
	New Hampshire DES	0.06	Chronic - carcinogen	PM4	Derived from ACGIH TLV-TWA of 25 ug/m3	Y
	New Jersey DEP	3	Chronic	PM4	Adopted CalEPA	N
	New York DEC	2	Chronic	PM4	Adopted TCEQ	N
	North Dakota DEQ	0.5	8-h	PM4	Derived from ACGIH TLV-TWA of 25 ug/m3	N
	Oregon DEQ	3	Chronic	PM4	Adopted CalEPA	N
	Texas TCEQ	0.27	Chronic - carcinogen	PM4	N/A	Y
	Vermont DEC	0.12	Chronic	NA	N/A	N
	Virginia DEQ	3	Chronic	Respirable	N/A	N
	Washington Department of Ecology	3	24-h	Respirable	N/A	N
Occupational exposure limits	CalEPA	50	PEL	PM4	8-h TWA	N
	Cal OSHA	50	PEL	PM4	8-h TWA	N
	ACGIH	50	TLV	PM4	8-h TWA	Y
	ACGIH	25	TLV-TWA	PM4	10-h TWA	Y
	OSHA	50	PEL	PM4	8-h TWA	Y
	MSHA	50	PEL	PM4	8-h TWA	Y
	NIOSH	50	REL	PM4	8-h TWA	Y

**Table 4:** Model predicted relative risk estimates for lung disease and associated descriptive statistics.

Outcome	Mean	n	Std	Lower CI	Upper CI
Silicosis	1.73	49	0.058706	1.71	1.75
Lung cancer	1.17	49	0.002315	1.169	1.171

For animal data PubMed literature searches for lung cancer benchmark dose models (BMD) and existing benchmark dose limits (BMDL) were conducted using search terms “benchmark dose modeling,” “BMD,” “*in vitro*,” “silica,” “SiO2,” and “cancer” or “lung cancer.” These results were cross-referenced with IARC (2) and ATSDR (1) for completeness.

### 2.4 Risk characterization methods

Traditional risk assessment methods were applied to the above reported study results. For cancer risk based on linear dose–response assumptions, theoretical risk estimates for ambient and background levels of RCS were calculated using the resulting occupational epidemiology-based regression equation after adjustment of input exposure doses for extrapolation to a community relevant exposure duration. For extrapolation from the animal data, exposure levels were standardized, and crude odds ratios (ORs) were calculated utilizing the observed incidence of lung cancer among exposed and unexposed rats, mice and hamsters from studies reported in (2). The assessment approach under the assumption of a threshold-like response is presented as a HQ. These methods are typical for EPA methods community risk assessment (46).

## 3 Results

### 3.1 Epidemiological analysis of RCS exposure levels and associated dose response

Meta-analyses and dose–response studies within our epidemiological review showed a clear dose–response relationship with increasing exposure resulting in increasing risk of silicosis. Generally, clear increased risk of silicosis were observed at lower exposure concentrations than for lung cancer. Several epidemiological studies showed a significant positive relationship between increasing silica exposure and lung cancer risk, with higher risks observed among specific silica polymorphs (22, 30, 47–59). Other studies have shown that reducing silica exposure from 0.1 mg/m^3^ to 0.05 mg/m^3^ may reduce associated lifetime risk of silicosis and lung cancer (5, 17, 60). Further, exposure-response relationships have been observed, with higher cumulative silica exposure associated with increased silicosis and potentially, lung cancer risk (61, 62). The mining industry and others in which the grinding or crushing of natural or engineered stone containing crystalline silica appear to have the highest risk among silica-exposed workers with the highest normalized RCS generation rate occurring between 3.2 and 5.6 μm (53, 63). There are significant databases of exposure information for silica associated with various industrial processes, such as the data published by OSHA from inspections and their special and national emphasis programs.^7^

However, other reviews reported the dose–response relationship between silica and lung cancer to be (1) unclear, (2) lacking in the absence of silicosis, or (3) confounded due to smoking (14, 29, 32–34, 36, 37). Indeed, in an exploratory study mathematical modeling silica-induced lung effects, the author noted that RCS exposure appeared to have a threshold-like behavior, below which the risk of lung cancer was not increased (19).

Meta-analyses and cohort studies have consistently shown positive associations, with risk ratios increasing primarily at higher exposure levels (55, 64). In one such study, the lifetime excess risk of lung cancer was estimated at 0.51% for workers exposed to the previous OSHA standard, 0.1 mg/m^3^ from ages 20–65 (62). Another study found that exposure also at the previous OSHA standard of 0.1 mg/m^3^ was associated with a 30% increased risk (60). Some studies reported significant dose–response trends even among non-silicotic workers (47, 49). However, the carcinogenic role of silica in the absence of silicosis remains unclear (21, 36).

#### 3.1.1 Collation of relevant fenceline exposure data and exposure limits

Mundt et al. (35) explored quantitative exposure-response results for occupational exposure to silica and associations with lung cancer. The authors utilized a hybrid systematic review approach including critical assessment of study quality based on guidance from US EPA’s TSCA and IRIS, and others. In total 20 studies that quantitatively addressed lung cancer and silicosis were systematically reviewed and the evidence synthesized. The authors concluded that the risk of lung disease was most clearly increased at high levels of cumulative exposure, with silicosis risk most clearly seen above 3 mg/m^3^-yrs. The authors demonstrated that lung cancer risk may not be increased until even greater exposure levels are achieved at which silicosis risk is also elevated (35, 65).

Most of the epidemiological research to date has focused on occupational populations and risk of silicosis, lung cancer or other diseases. However, the potential lung cancer risk related to ambient RCS exposure have received less study. Occupational silicosis is well-documented, and lung cancer risks have been reported in some studies such as among underground miners who also were exposed to diesel fumes and radon (66). However, the assessments regarding ambient silica exposure risks are less well studied and also warrant attention (42, 43). For exposure to communities adjacent to sand, stone, and gravel operations specifically, the level of exposure lies somewhere between modest additions to background levels and the low end of occupational exposure ranges (39). Regulatory agencies, state agencies, and primary literature report background ranges of silica between 0 and 3 μg/m^3^ with no silica related lung effects apparent [Table 1; (39)].

A challenge in assessing community risk lies in the relatively low exposures to RCS and the relatively high background incidence of lung cancer. According to SEER (67) lung cancer incidence in the general population is approximately 49 per 100,000 men and women per year (67), in large part driven by the very high incidence rate among active smokers, e.g., 172 per 1,000 current smokers as estimated by Villeneuve and Mao (68). Thus, any small increase in RCS related exposure risks relevant to risk assessment levels of concern for chemical exposures (in the range of 10^−4^ to 10^−6^ additional risk) would not be directly detectable in a community study. An alternative approach is to use the occupational epidemiology data as a surrogate for community risk assessment and use such data to derive a linear relationship between exposure and risk. However, the linear dose response derived from occupational silicosis cannot be correct according to Haber’s rule which states that the severity of a toxic effect is directly proportional to the product of the concentration of a toxic substance and the duration of exposure to that substance (C x T). Therefore silicosis (and possibly lung cancer) would be identified within the general population based on the incidence predicted by such equations (Equation 2), if the traditional linear-dose response were correct.

Therefore, community-level exposures to silica are unlikely driving lung cancer incidence rates and the traditional risk assessment approach of the linearization of the occupational dose response data (46) likely is inappropriate. From previous reviews, the cumulative exposure threshold for excess risk of silicosis in occupational groups appears to be roughly 3 mg/m^3^-yrs (35) and roughly 10 mg/m^3^ -yrs for lung cancer, although one study estimated that lung cancer risks began to increase among those exposed above 2.4 mg/m^3^-yrs (based on an open-ended highest exposure category) among never smokers (52). Converting the cumulative exposure among never smokers at which lung cancer began to increase (2.4 mg/m^3^-yrs) to air concentrations, and assuming a 45-yr working lifetime, this potential threshold is equivalent to a workplace air concentration^8^ of 53 μg/m^3^. This potential threshold estimated from the cumulative increased risk for silicosis reported by Mundt et al. (35) of 3 mg/m^3^-yrs is generally consistent with the range of occupational onset effect doses for silicosis published by various occupational health agencies (4, 69) and reviewed in Patty’s 2024. Specifically, these reviews describe onset doses for silicosis as the following:

- OSHA (4) reported an estimated threshold effect for inflammation and carcinogenesis of 36 μg/m^3^ over a 45-year working lifetime. The OSHA action level is 25 μg/m^3^ and the PEL is 50 μg/m^3^.
- Patty’s Silica Toxicological Chapter (69) reports that there is consensus that reducing the percentage of respirable free silica reduces the incidence of silicosis and recommends maintaining exposures below the current PEL of 50 μg/m^3^ for an 8-h TWA workday over a 40-h workweek.

Extrapolating this concentration to a general population community level equivalent^9^ on the basis of duration of exposure differences, suggests the onset of silicosis and perhaps cancer risk could be expected at concentrations as low as 4 μg/m^3^. This is close to the California Environmental Protection Agency’s general population Office of Environmental Health Hazard Assessment Reference Exposure Level (OEHHA REL) of 3 μg/m^3^. Thus, when conducting a traditional risk assessment, the data suggest that the onset dose for silicosis and possibly lung cancer is close to the range of community level exposures (Table 1). This results in an extremely small margin of safety (MoS), and it is more likely than not that the onset dose for lung effects is much higher than the range derived from the linear extrapolation model for community exposures. Therefore, the challenge of a traditional risk assessment is that it results in an inaccurate or oversimplified conclusion. However, silicosis is historically an occupational disease, not observed in the general population, and lung cancer risks among the general population surrounding sand and gravel facilities are not known to be elevated.

To explore the potential difference between silicosis and lung cancer risks at the general population level, a simple linear regression for lung cancer risk and cumulative exposure to RCS from the empirical data was performed in RStudio. This approach reflects a screening assessment given the uncertainty of the data and questions about the dose response for cancer. A linear approach is often used as a default for cancer assessment when the MOA is not sufficiently demonstrated to support a threshold-like response model. Simple linear regression initially resulted in an equation of the form:


(1) $$\text{Lung Cancer Risk}=0.069(\frac{\text{mg}}{m^{3}})X+1.17,p=0.017$$

The effect modifier was statistically significant (*p* = 0.017), however, overall, the line showed poor fit (adjusted R^2^ = 0.28) (Equation 1).

Likewise, simple linear regression for silicosis risk and cumulative exposure to RCS resulted in a regression equation of the form:


(2) $$\text{Silicosis Risk}=1.75(\frac{\text{mg}}{m^{3}})X+1.67,p<0.001$$

**Figure 1:**
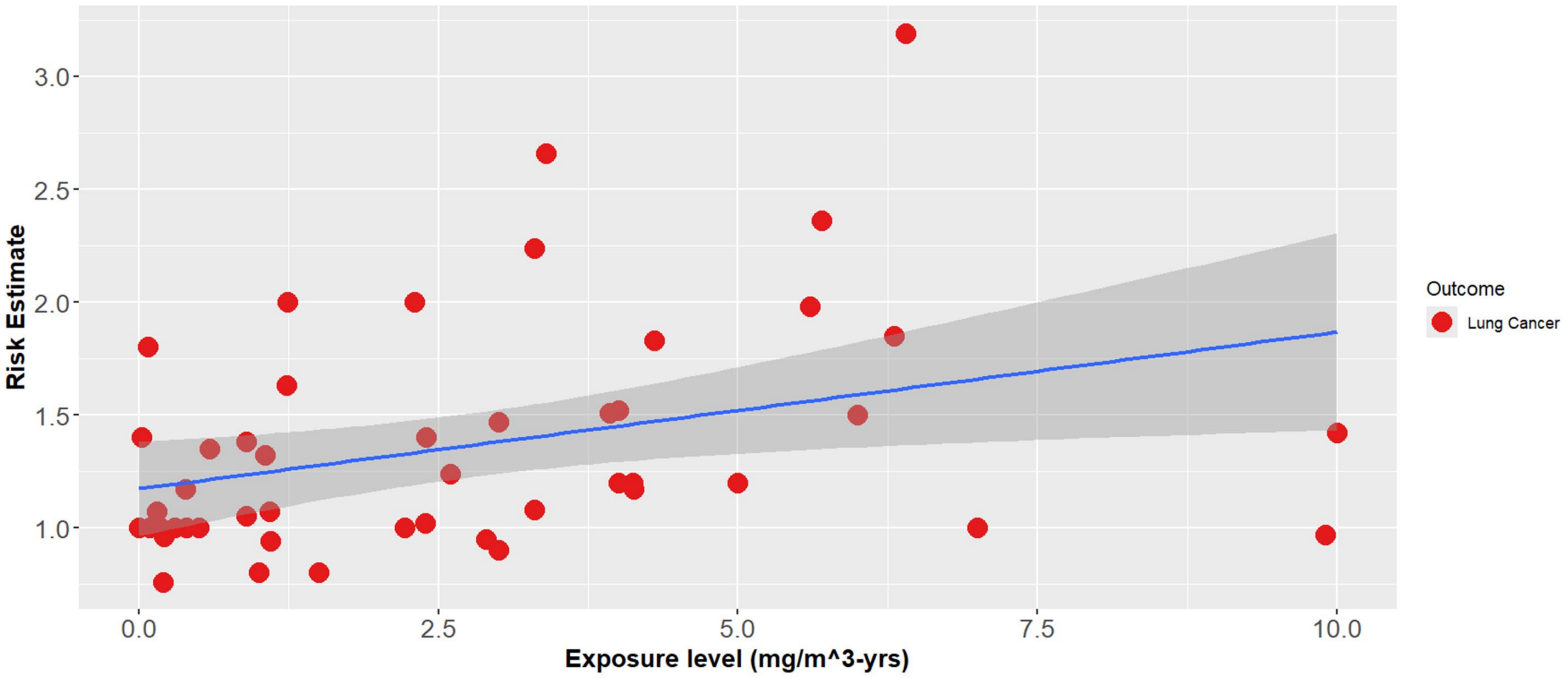
Risk of lung cancer and silica exposure levels from occupational epidemiology.

**Figure 2:**
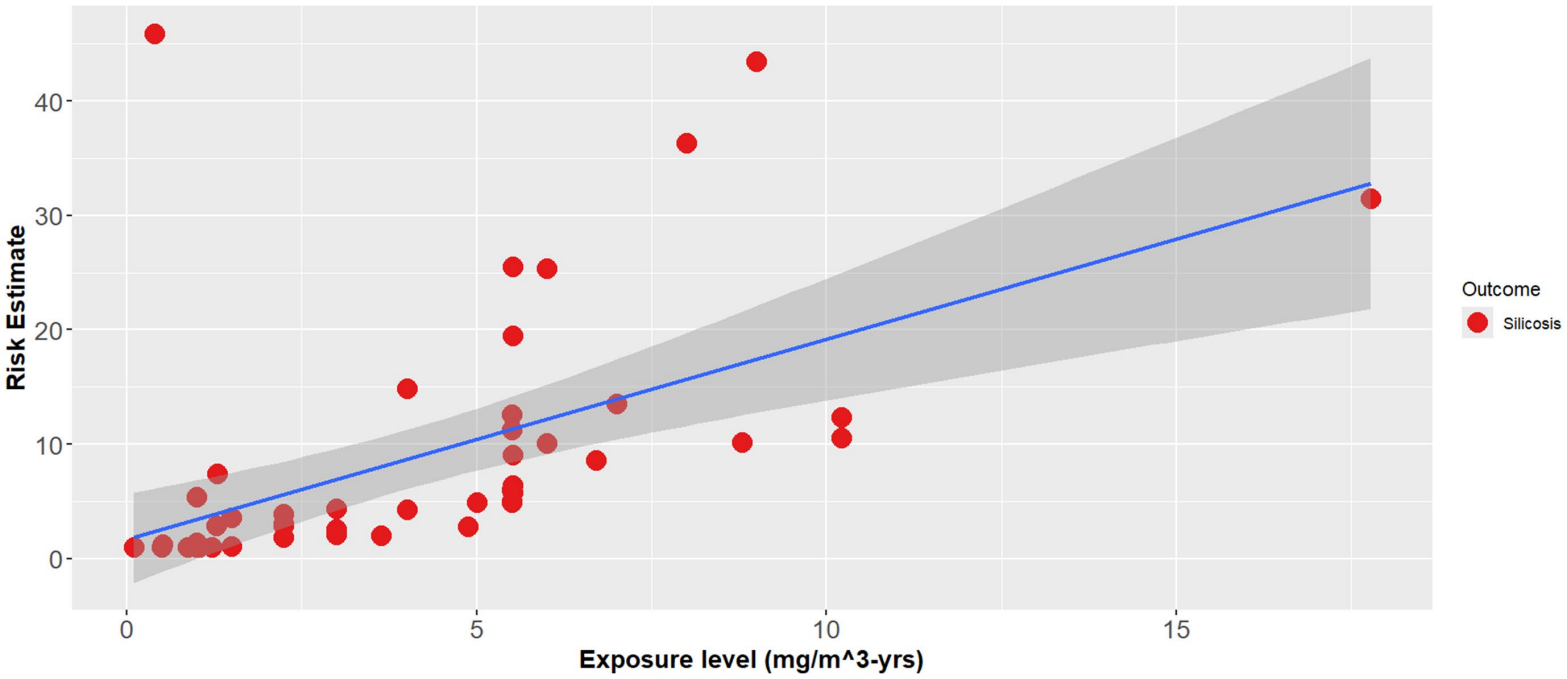
Risk of silicosis and silica exposure levels from occupational epidemiology.

**Figure 3:**
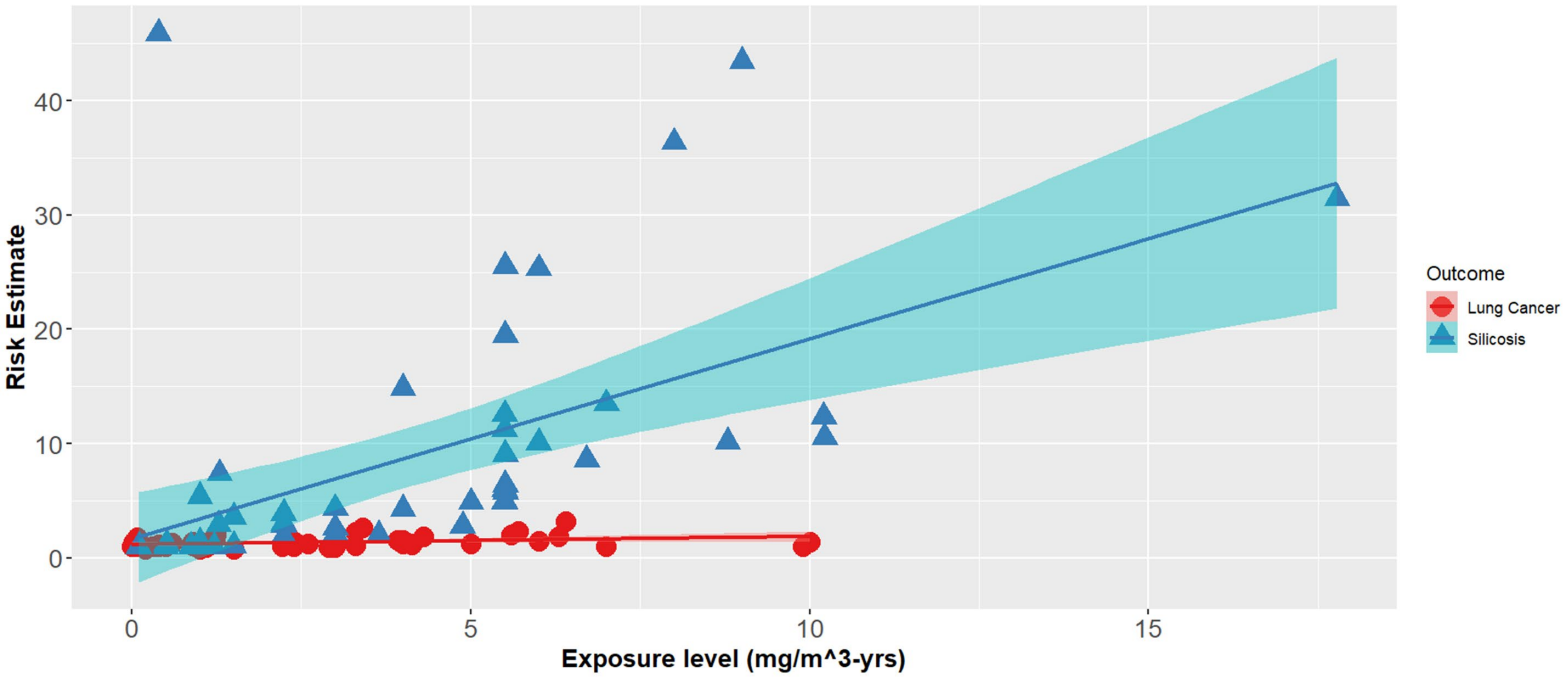
Combined lung disease risk estimates and silica exposure levels reported in the epidemiological literature.

The effect modifier was also statistically significant (*p* < 0.001), however the adjusted R^2^ also showed a poor regression line fit (adjusted R^2^ = 0.1) (Equation 2). Figures 1–3 demonstrate the relative relationships between lung disease and silica exposure levels.

Figure 1 illustrates the risk of lung cancer at exposure levels reported in the epidemiological literature. Specifically, risk estimates ranged from below unity to as high as 3.25 for exposure levels greater than 5.0 mg/m^3^-years. Figure 2 illustrates the risk of silicosis at exposure levels also reported in the epidemiological literature. Notably, the risk of silicosis was about one order of magnitude higher than the risk of lung cancer risk at every level of exposure (mg/m^3^-yrs). Further, Figure 1 illustrates that lung cancer risk does not become significantly greater than unity until exposure levels at which silicosis risk is also significantly elevated, i.e., 5 mg/m^3^-yrs (Figures 1, 2). Additionally, when combining both lung cancer and silicosis risk, it becomes clear that exposure levels reported in the epidemiological literature are more highly associated with silicosis rather than lung cancer and it appears that our regression analysis demonstrates a different, and significantly higher onset dose for lung cancer than that of silicosis (Figure 3).

Ambient silica levels reported in Richards and Brozell (39) (Table 1; (39), Table 3.8) were averaged and resulted in an average urban RCS concentration of 0.000421 mg/m^3^. This exposure level was then converted to an exposure range in mg/m^3^-years accounting for the average working year and working conditions: assuming 270 working days of exposure for every 365 days, 10 m^3^ volumetric exposure for every 20 m^3^ and 30 years working history equivalent, and assuming 365 days of exposure over 70 years, which resulted in the following equation:


(3) $$70\;\text{yrs}\times 0.000421\frac{\text{mg}}{m^{3}}=0.0295\;\frac{\text{mg}\;x\;\text{yrs}}{m^{3}}$$

Therefore, the community air concentration calculated as an equivalent workplace cumulative dose = 0.0295 mg/m3-yrs (Equation 3). This value can be used in the regression equations that were based on occupational epidemiology to determine the estimated increase in risk for an equivalent exposure concentration as occurs in the community.

The regression equations on the ambient silica levels reported in Richards and Brozell (39), resulted in a risk estimate of 1.73 (95% CI: 1.71, 1.75) for silicosis and a risk estimate of 1.17 (95% CI: 1.169, 1.171) for lung cancer. However, TCEQ notes that there “is no federal regulation or EPA standard for ambient crystalline silica concentrations, and there is no EPA requirement for [states] to monitor for [ambient] crystalline silica” suggesting that there does not appear to be an urgent need to establish a sampling requirement, since silica is a known and prevalent material.

### 3.2 Mode of action (MOA) analysis

Lung cancer risk may not be associated with exposure levels below those at which silicosis risk is elevated (35). Furthermore (19) demonstrated the mode of action for silica induced lung effects appears to be threshold-like. For that reason, there likely is a level of exposure at which no adverse lung effects would be expected to be observed. To illustrate the uncertainty between community level exposures and lung disease RCS, at low doses of RCS, it becomes imperative to understand the similarities and differences in the MOA for silicosis and lung cancer. Silicosis itself is not observed in the general population. Based on the analysis of the epidemiology, it is a more sensitive effect and can serve as a protective biomarker surrogate for assessing lung cancer risk. This approach is needed as silicosis is a specific disease associated with RCS exposure, while lung cancer is not.

**Figure 4:**
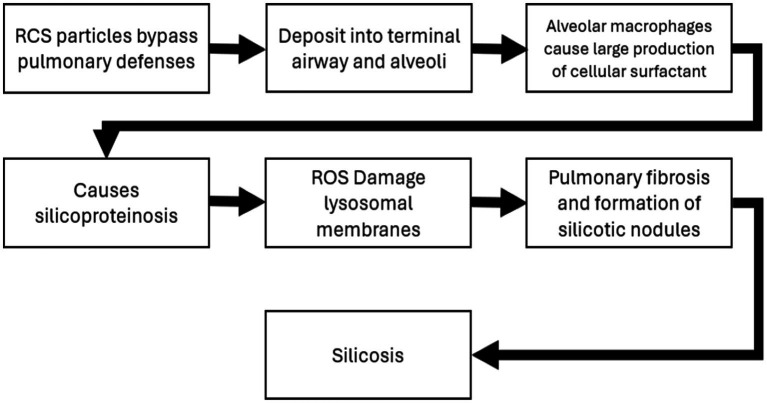
Adverse outcome pathway and mode of action for silicosis (71).

**Figure 5:**
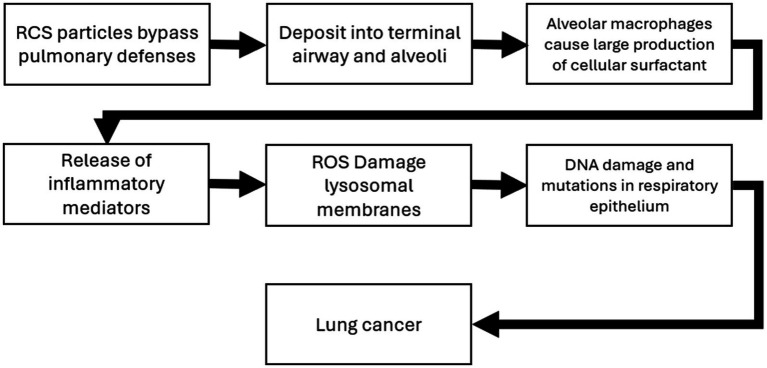
Adverse outcome pathway and mode of action for lung cancer (71).

Chronic silicosis has also been associated with a higher risk of lung cancer in animal models (17). The carcinogenic potential of silica is supported by both animal studies and epidemiological evidence (1, 2, 17, 70). The mechanisms of silica induced silicosis involve direct cytotoxicity, oxidative stress, inflammation, and fibrosis (15, 16). Specifically, silica particles generate reactive oxygen species, leading to cell damage and activation of inflammatory pathways (84). While some studies suggest a direct link between silica exposure and lung cancer, others propose that silicosis is a necessary intermediate step (14). Adverse outcome pathways (AOP) detailing the potential MOA for both silicosis and lung cancer have been developed and are summarized in Figures 4, 5 (2, 71). The foundational mechanism causing the onset of inflammation is still an area of active research although recent progress with additional hypothesis has been made (69). Briefly, RCS is believed to have direct cytotoxicity based on what is known about its silonal surface chemistry (10, 72, 73). The resultant stimulation of alveolar macrophages causes recurrent lung damage and result in inflammatory markers which activate polymorphonuclear leukocytes causing further lung damage (71, 73). The stimulation of alveolar macrophages induces fibroblast proliferation and stimulate collagen synthesis which causes pulmonary fibrosis and silicosis (71, 73).

Several meta-analyses have found an elevated risk of lung cancer among silicosis patients, even after adjusting for smoking (22, 52). Smoking significantly increases lung cancer risk in silica-exposed workers, with a possible multiplicative interaction (29, 52, 61). However, the relationship between silica exposure, silicosis, and lung cancer remains complex, with potential confounding factors and methodological challenges in epidemiological studies (23, 70). Therefore, it would appear that the MOA is a parallel process for silicosis and lung cancer with both oxidative stress and inflammation appearing to be key events. The epidemiological evidence described above also demonstrates that silicosis is the more sensitive adverse effect with a lower threshold and higher prevalence at specific quantified occupational exposure levels of RCS (73). Therefore, preventing silicosis would appear to also prevent lung cancer at all relevant levels of exposure. A proposed AOP for lung cancer is described below. These MOA hypotheses support a threshold-like dose–response approach, although a non-threshold linear low-dose response is hypothetically possible. A threshold-like approach appears to have been used for existing community airborne limits. However, our cancer screening approach above (section 3.1.1) did incorporate a linear dose–response methodology.

As outlined in Table 3, several state agencies have developed community health-based exposure limits (although the majority do not specifically denote that they account for cancer effects Table 3). The Minnesota DPH derived a particularly transparent chronic health-based value (HBV_chronic_), similar to the current analysis, of 3 μg/m^3^ and a point of departure (POD) of 0.0098 mg/m^3^ (Minnesota DPH, Table 3).

### 3.3 *In vivo* dose–response evidence

Dose–response and benchmark dose modeling via *in vivo* studies of RCS and associated lung cancer were limited. Similarly, agency reviews (1, 2, 21) report only single-dose inhalation studies which precluded estimating an adequate dose-response. These studies are summarized in Table 5. Briefly, results were mixed for rats with several studies showing statistically significant induction of tumors following various doses of RCS (24–27). However, one study in rats and one study in hamsters did not show statistically significant tumors in rodents exposed to RCS (28). A single genotoxicity *in vivo* study was positive for hprt-locus gene mutation in alveolar type II epithelial cells (74). Additionally, a separate review by OEHHA (75) identified six rodent studies previously analyzed by EPA (91) that derived lowest observed adverse effect levels (LOAELs) for silica effects ranging from 0.2 mg/m^3^ to 4.9 mg/m^3^ (75).

**Table 5:** Animal data [adapted from (2)].

Species, strain (sex)	Exposure duration	Dosing regimen	Respiratory tumor incidence	References
Mouse, BALB/C BYJ (F)	150, 300, or 570 d	0, 1.5, 1.8 or 2.0 mg/m3 8 h/d, 5 d/wk	7/59 (control), 9/60 (all exposed)	(28)
Rat, F344 (M, F)	24 months	0,52 mg/m3 6 h/d, 5d/wk	M-0/42 (control), 1/47\u00B0F-0/47 (control), 10/53	(24)
Rat, F344 (F)	Lifespan	0,12 mg/m3 6 h/d, 5d/wk. for 83 wk	0/54 (control), 18/60	Holland et al. (89, 90) (25)
Rat, SPF F344 (M, F)	30 mo	0,1 mg/m3 6 h/d, 5 d/wk. for 24 mo	3/100 (control M, F), 7/50 (M), 12/50 (F)	(69) Muhle et al. (87, 88)
Rat, Wistar (F)	up to 35 mo	0,6.1, 30.6 mg/m3 6 h/d, 5 d/wk. for 29 d	0/85 (control), 37/82 (low dose), 43/82 (high dose)	(27)

**Figure 6:**
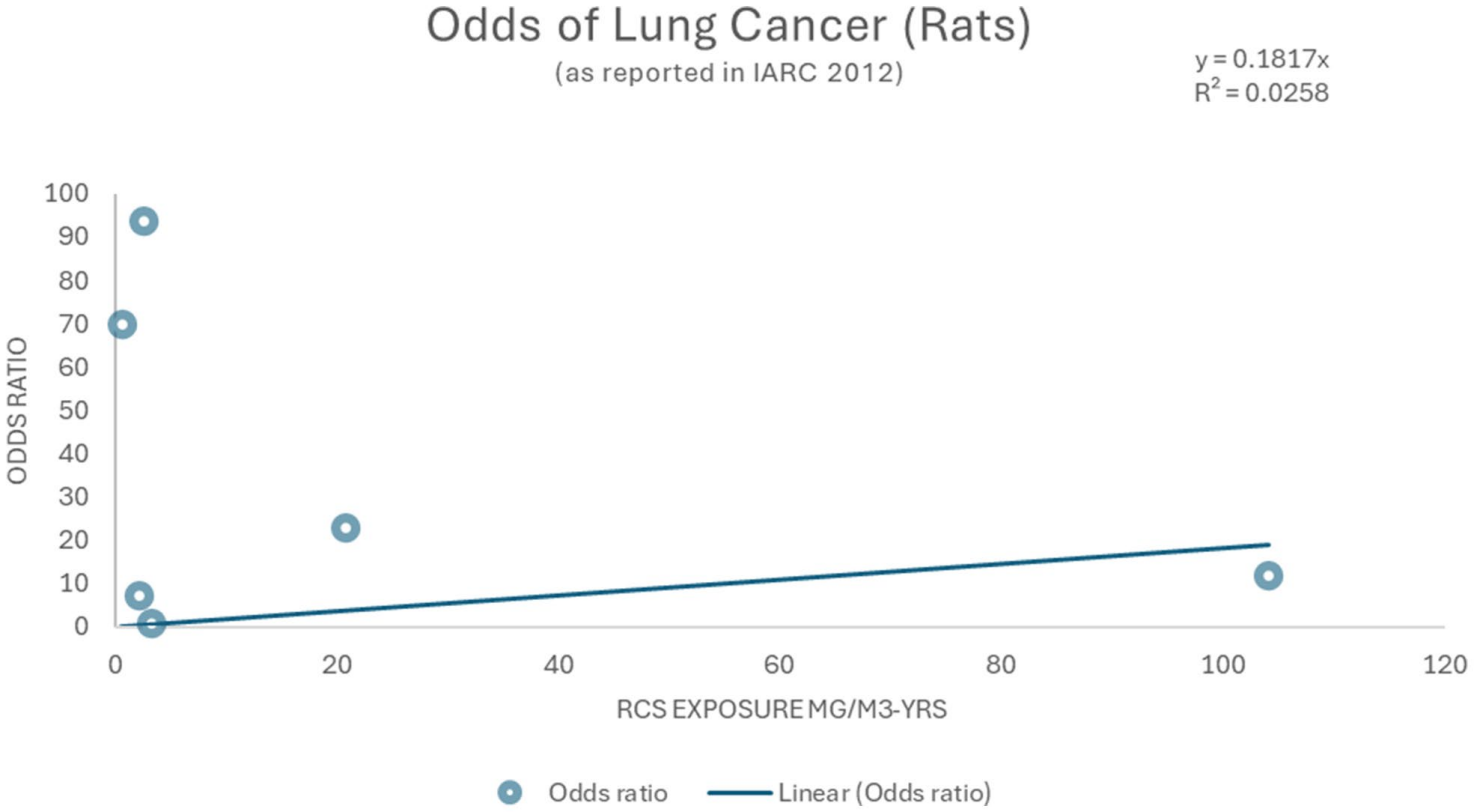
Linear model and Odds ratios for lung cancer (2).

As shown in Figure 6 the data do not provide clear evidence of a quantitative dose–response relationship for lung cancer. Using the inhaled silica exposed rodent studies reported by IARC (2), crude odds ratios for lung cancer were calculated and ranged from 1.21 to 93.72 (Figure 6) but cannot be used with reasonable confidence given the imprecision indicated by the wide spread of the data and crude (i.e., unadjusted) ORs.

One benchmark dose study on human A549 lung cells derived a BMDL for 24-h treatment of C-SiO2 MPs and -NPs to be 2.26 and 0.97 μg/mL and 1.17 and 0.85 μg/mL for 72-h treatment, respectively (11). The authors concluded that particle size remained a contributing factor when assessing human health occupational risks as other studies had shown that altering particle size may impact toxicity (11). However, translation of these to a relevant equivalent air-concentration cannot be done with confidence due to limitations of *in vitro* to *in vivo* dose extrapolations for respiratory effects (76).

### 3.4 Risk characterization and hazard quotient

Richards and Brozell (39) reported average ambient RCS measurements from a variety of urban settings. The author compared the ambient RCS concentrations to the OEHHA REL of 3 μg/m^3^ and observed that all measurements were significantly below this regulatory exposure limit. Complete results can be found in Table 3.8, however the average across all sites was 0.000421 mg/m^3^. The resultant HQ (0.421/3 μg/m^3^) is 0.14. This is 7 times below the current general population equivalent of occupational exposure levels (as demonstrated in section 3.1.1). However, when compared to the lowest community health-based exposure limits (0.27 μg/m^3^) an even smaller margin of safety (HQ of 1.56 which exceeds the target of unity) is achieved. This analysis alone would indicate that ambient exposure levels surrounding sand, stone and gravel facilities are likely within an adequate margin of safety for the general population in most cases, but the upper exposure range and the lower range of exposure limits suggest additional assessment is needed. However, Puledda et al., measured urban RCS and found PM4 RCS concentrations ranged from 0.6–1.5 μg/m^3^, and Davis et al., measured urban RCS and found concentrations ranged from 0.9–1.9 μg/m^3^. These measured ambient exposures are below some but not all regulatory limits (Tables 1, 3). Thus, using the HQ approach and current community limits as the basis for risk assessment indicates that additional analysis is needed because some of the HQ calculation scenarios exceeded unity and suggest small margin of safety (MOS). Thus, similar to the analyses using traditional linear methods, this risk characterization result does not align with the absence of observed silicosis or lung diseases from general ambient exposures.

### 3.5 Summary of key results

1. The most robust epidemiological studies tend not to show an increased risk of lung cancer at exposure levels below those at which silicosis risk is clearly increased.
2. Because the default linear approach to the cancer data is precautionary, applying this approach may inappropriately raise concern for increased risk of lung cancer from community or fence line – or even ambient RCS exposures.
3. Using threshold dose–response approaches and by adjusting occupational limits for non-cancer effects also gives the misleading impression of a potential lung cancer risk for community exposures.
4. The health surveillance data (i.e., the “real world” observations) do not align with or substantiate these potential community level exposure risk characterization results, suggesting that classic default methodologies using occupational potency data are inadequate for deriving valid estimates of community risks of silicosis and lung cancer due to ambient concentrations of RCS.
5. Further refinement of quantitative risk assessment methods based on MOA principles is needed to develop risk assessment approaches that generate accurate risk estimates for silicosis and lung cancer for community RCS exposures with greater confidence.

## 4 Discussion

Our analysis illustrated that extrapolating community level risk from the occupational data suggests that community exposures exceed the carcinogenic threshold. However, from observational data we do not see an increased risk of RCS-induced lung cancer or silicosis in the general population (35). From our analysis it appears that to adequately assess risk of silicosis and lung cancer, if any, due to community level exposure to RCS, we first need to address why there are no observable increases of these diseases associated with even low to moderate occupational exposures (i.e., up to current occupational exposure limits). Primarily community exposures do not exceed the threshold necessary for carcinogenesis to observe elevated levels of lung cancer. Further, silica itself may not cause lung cancer except at exposures exceeding 3 mg/m^3^-years. The evidence indicates that silicosis can serve as a sensitive marker for RCS induced lung effects, and preventing silicosis would theoretically eliminate lung cancer risk. Therefore, silicosis may be treated as a specific marker for risk when analyzing exposures to silica and assessing the possibility of lung cancer risk. From epidemiological reviews, overall lung cancer in the general population (i.e., non-occupational population) is either zero or too small to detect to come to any conclusion on the causal role of background level exposures of silica. Therefore, we may assume, if silicosis is not observed in the general population, then there would not be a later related onset of lung cancer. Further, background or ambient levels of silicosis can be analyzed and measured with a reasonable degree of certainty in the general population.

Our analysis is not without limitations. Primarily we did not conduct a site-specific risk assessment but relied on exposure measurements from a wide range of extraction facilities as reported in Richards and Brozell (39) and the observational epidemiology from Mundt et al. (35) to present a general non-site specific risk characterization compared to observations in the general population. Site specific risk assessments would benefit from specific statistical analyses that were outside the scope of this work, including GIS-based dispersion modeling, Monte Carlo simulation to address variability, comparison to U.S. national/regional cancer registries for baseline risk, and accounting for lung cancer latency through lagging algorithms.

Reviews of the body of evidence demonstrating that RCS causes silicosis, and possibly lung cancer, vary widely according to exposure circumstances (13, 44, 65, 72, 77, 78). These reports support targeting regulatory activities to industries and processes with the highest likelihood of occupational exposure to RCS—where silicosis (and possibly lung cancer) can be prevented—rather than the general background community, where ambient exposures, including those in the vicinity of sand and gravel extraction facilities, remain well below any exposure threshold for risk of these diseases. Thus, current risk assessments for communities rely on occupationally derived dose–response data. Such data may not be representative of the effects of RCS from community exposure. Specifically, no studies were identified which investigated occupational versus community related factors such as differences in dosimetry/deposition, intensity, and potency of fractured silica particles and the role they play in contributing to the likelihood of lung diseases (including cancer). This illustrates a need for further investigation of such factors to better understand population level risk from ambient RCS levels.

### 4.1 Three factors that influence the disease-causing potential of RCS

#### 4.1.1 Inhalation dosimetry/deposition

As demonstrated in quantitative ambient RCS measurements (39) workers may have a higher dose of RCS normalized to exposure concentration, in general, and per exposure event based on particle size characteristics and crystalline silica content of different particles. This leads to a higher overall exposure concentration of RCS coupled with a higher breathing rate and depth, positional factors, and particle distribution differences within occupational settings. Furthermore, studies have shown that altering particle size can impact particle toxicity (11, 79, 80). As stated previously, occupational, and ambient RCS exposure can contribute to respiratory diseases. However, particle size, itself has been shown to be a crucial factor in disease outcomes (40).

One tool to account for particle size and distribution is inhalation dosimetry. From first principles, workers are exposed to smaller, freshly fractured particles (higher respirable content) and breathing rates are higher, due to strenuous labor activities. In theory, any delivered RCS dose would be higher for workers versus the general population. There are a variety of modeling tools to provide adequate comparisons between the occupationally exposed and those in the general population in terms of deposited RCS dose. Such methods can be used to adjust occupational point of departure doses to general population equivalent doses for improved risk estimation.

#### 4.1.2 Exposure intensity (likelihood for and impact of periods of intense exposure)

Due to differences in intensity, the same cumulative exposure may cause different risks increases in occupational and general population settings. It is well known from occupational scenarios that dose rate (intensity) impacts silicosis risk and progression. However, data are lacking on precise estimates of dose-rate versus risk estimates. Further, the complications of irregular exposure periodicity on risk are not quantitatively understood. However, what is currently hypothesized is that peaks in exposure in addition to overall cumulative dose could cause different outcomes versus exposures at only low doses at a steady dose rate. Workers in occupational settings can experience peaks in exposure whereas exposure changes experienced by the general population are smoother or relatively constant. Thus, use of occupational data might overestimate potency compared to community exposures due to the impact of peak exposure periods. This hypothesis needs additional examination for adjusting community risk assessment findings, but as outlined in Table 6 the lifetime risk of silicosis varies under different exposure frequencies with extreme exposure conditions exhibiting the greatest percent increase in silicosis risk (25% vs. 47% lifetime risk increase) (4).

**Table 6:** Lifetime risk increase of silicosis and frequency of exposure.

Lifetime risk of Silicosis based on Intensity and frequency of exposure (4)		
Exposure level	Years exposed	Lifetime % risk increase
0.1 mg/m^3^	30 yrs	25%
0.09 mg/m^3^	45 yrs	47%
1–5 mg/m^3^	Per year	32%

#### 4.1.3 Relative potency (presence of free radical persistence)

The cancer potency of silica has been evaluated using both animal and human data, with human epidemiological studies providing more reliable potency-corrected risk assessments (81). Different silica types exhibit varying potencies in stimulating arachidonic acid metabolism and cytotoxicity in alveolar macrophages (82). Current experimental evidence indicates that crystalline silica varies in toxicity according to the specific polymorphic form (44).

Further, additional studies have shown varying RCS potency under different experimental conditions (10, 77, 78). Bellomo et al. (10) investigated the membranolytic activity of quartz in different molecular environments and found that the type and reactivity of radical sites on quartz were influenced by the outer molecular environment (10). Vallyathan et al. (77, 78) demonstrated that freshly fractured quartz had increased surface activity and caused greater inflammatory markers than aged quartz (77, 78). In rat macrophages, freshly fractured quartz was found to be over four times more potent than silica aged 1–2 days (77). Similarly Vallyathan et al. (78) found that markers of lung injury, lipid peroxidation, and increased oxygen radicals were statistically significantly more pronounced in Fischer 344 rats exposed to freshly fractured quartz than those exposed to aged quartz (78). However, the long-term carcinogenic effect of such quartz in humans is less studied. This demonstrates that the biological activity of milled quartz may be dependent on particle size and environmental factors. Other studies have demonstrated that it is possible to measure biological activity *in vitro* of various aerosols including quartz using macrophage assay’s such as NR8383, providing a template to assess the potency of freshly fractured versus weathered silica (13).

Additional research can address the hypothesis that the types of emissions adjacent from to sand, stone and gravel facilities may have a different potency than the traditional occupational epidemiology experience. Therefore, extrapolating from the occupational epidemiology data is not scientifically rigorous as it is clear from the observational epidemiology that community versus occupational groups have different risk profiles (35). Potency also may vary between different occupational sources. These different sources may also include industries not included in this analyses such as construction sites, farming, and carpentry.

### 4.2 Future dose response options

Future dose response options can address the modifying factors identified through our current analysis. Some approaches to building a biologically based dose–response model include (1) categorical regression on identified key biological events (2) network mathematical modeling for lung cancer thresholds and (3) a modified HQ approach advancements in Bayesian mathematical models and categorical regression to identify key biological events for lung effects (19, 86) utilize empirical data to build biologically based dose–response models and identify lung cancer thresholds. One such study indicated that mathematical modeling of inflammation mediated lung diseases showed an exposure-response threshold below which, lung disease risk was not increased (19). Empirical data including ambient silica measurements, MIE identification, and differences between occupational and ambient levels of RCS exposure should continue to be explored to further develop these methods.

In the absence of such data, our current analysis supports a modified HQ or MOS modifier approach. Assuming the upper end of exposure distribution for the general population is 0.05 mg/m^3^ and the duration adjusted POD for silicosis is 0.0098 mg/m^3^ (Minnesota DPH, Table 3), then the resultant simple HQ is 5 for silicosis. However, when accounting for the three modifying factors proposed above [Dosimetry (D), Intensity (I), and Potency (P)] the actual community health-based HQ would be 0.05/ [0.0098 *(D*I*P)]. The inclusion of these factors, that all suggest community exposures would generate reduced risk versus occupational exposures. Future analyses will seek to quantify the 3 proposed modifying factors. Theoretically, this would result in a significantly reduced HQ which more accurately reflects the actual lack of observable silicosis and silica induced lung cancer in the general population.

## Data Availability

The original contributions presented in the study are included in the article/supplementary material, further inquiries can be directed to the corresponding authors.

## Footnotes

^1^Department of Labor issues final rule reducing silica dust exposure, better protecting miners’ health from irreversible workplace illnesses | U.S. Department of Labor.

^2^Silica and Worker Health | Silica | CDC.

^3^Community Public Health | US EPA.

^4^Community Public Health Fact Sheet | US EPA.

^5^Environmental public health research at the U.S. Environmental Protection Agency: A blueprint for exposure science in a connected world | Journal of Exposure Science & Environmental Epidemiology.

^6^Environmental Justice | US EPA.

^7^ https://www.osha.gov/laws-regs/standardinterpretations/1998-03-27

^8^Workplace air concentration: 2.4 mg/m^3^ -yrs * 1000 ug/mg *1/45-yrs = 53.3 ug/m^3^.

^9^General population equivalent of occupational exposure levels: 53 μg/m^3^ * 45 yrs./70 yrs. * 5 days/7 days * 10 m^3^/20 m^3^/3 UF. (3 UF: Uncertainty factor accounting for general population vs. worker susceptibility).

## References

[1] Agency for Toxic Substances and Disease Registry (ATSDR). Toxicological profile for Silica. Atlanta, GA: U.S. Department of Health and Human Services, Public Health Service (2019). 37410876

[2] IARC. Silica dust, crystalline, in the form of quartz or cristobalite. IARC monographs on the evaluation of carcinogenic risks to humans. Volume 100C. Arsenic, metals, fibres, and dusts. Lyon, France: International Agency for Research on Cancer (2012). PMC4781271 23189751

[3] Misra S Sussell AL Wilson SE Poplin GS. Occupational exposure to respirable crystalline silica among US metal and nonmetal miners, 2000–2019. Am J Ind Med. (2023) 66:199–212. doi: 10.1002/ajim.23451, PMID: 36705259 PMC11146836

[4] OSHA (2016). OSHA’s respirable crystalline silica standard for construction.

[5] Howlett P Gan J Lesosky M Feary J. Relationship between cumulative silica exposure and Silicosis: a systematic review and dose-response Meta-analysis. Thorax. (2024) 79:934–42. doi: 10.1136/thorax-2024-221447, PMID: 39107111 PMC11503121

[6] Shao Y Almberg KS Friedman LS Cohen RA Go LHT. Thin seams and small mines are associated with higher exposures to respirable crystalline silica in US underground coal mines. Occup Environ Med. (2024) 81:308–12. doi: 10.1136/oemed-2023-109347, PMID: 38937079

[7] Albin M Gustavsson P. A silent epidemic: occupational exposure limits are insufficiently protecting individual worker health. Scand J Work Environ Health. (2020) 46:110–2. doi: 10.5271/sjweh.3864, PMID: 31720691

[8] Keil AP Richardson DB Westreich D Steenland K. Estimating the impact of changes to occupational standards for silica exposure on lung Cancer mortality. Epidemiology. (2018) 29:658–65. doi: 10.1097/EDE.0000000000000867 29870429 PMC6066423

[9] Shi T Zhang H Zhao M. Does the current occupational exposure limit effectively prevent the risk of silicosis? Thorax. (2025):24:223167. doi: 10.1136/thorax-2025-223167 40274411

[10] Bellomo C Lagostina V Pavan C Paganini MC Turci F. Reaction with water vapor defines surface reconstruction and Membranolytic activity of quartz milled in different molecular environments. Small. (2024) 20:e2308369. doi: 10.1002/smll.202308369, PMID: 38102095

[11] Rafieepour A Shekarloo MV Ashtarinezhad A Alimohammadi I Panjali Z. Benchmark dose determining airborne crystalline silica particles based on A549 lung-cell line survival in an in vitro study. Sci Rep. (2024) 14:21458. doi: 10.1038/s41598-024-72607-5, PMID: 39271741 PMC11399125

[12] Rao KM Porter DW Meighan T Castranova V. The sources of inflammatory mediators in the lung after silica exposure. Environ Health Perspect. (2004) 112:1679–85. doi: 10.1289/ehp.7295, PMID: 15579413 PMC1253659

[13] Wiemann M Vennemann A Sauer UG Wiench K Ma-Hock L Landsiedel R. An in vitro alveolar macrophage assay for predicting the short-term inhalation toxicity of nanomaterials. J Nanobiotechnol. (2016) 14:16. doi: 10.1186/s12951-016-0164-2, PMID: 26944705 PMC4779246

[14] Brown T. Silica exposure, smoking, silicosis and lung cancer—complex interactions. Occup Med. (2009) 59:89–95. doi: 10.1093/occmed/kqn171, PMID: 19233828

[15] Castranova V. From coal mine dust to quartz: mechanisms of pulmonary pathogenicity. Inhal Toxicol. (2000) 12:7–14. doi: 10.1080/08958378.2000.11463226, PMID: 26368596

[16] Rimal B Greenberg AK Rom WN. Basic pathogenetic mechanisms in silicosis: current understanding. Curr Opin Pulm Med. (2005) 11:169–73. doi: 10.1097/01.mcp.0000152998.11335.24, PMID: 15699791

[17] Sato T Shimosato T Klinman DM. Silicosis and lung Cancer: current perspectives. Lung Cancer. (2018) 9:91–101. doi: 10.2147/LCTT.S156376, PMID: 30498384 PMC6207090

[18] Shi X Ding M Chen F Wang L Rojanasakul Y Vallyathan V. Reactive oxygen species and molecular mechanism of silica-induced lung injury. J Environ Patho Toxicol Oncol. (2001) 20:10–93. doi: 10.1615/JEnvironPatholToxicolOncol.v20.iSuppl.1.80, PMID: 11570677

[19] Cox J Anthony L. An exposure-response threshold for lung diseases and lung Cancer caused by crystalline silica. Risk Anal. (2011) 31:1543–60. doi: 10.1111/j.1539-6924.2011.01610.x, PMID: 21477084

[20] Möhner M Nowak D. Estimation of an exposure threshold value for compensation of silica-induced COPD based on longitudinal changes in pulmonary function. Int J Environ Res Public Health. (2020) 17:9040. doi: 10.3390/ijerph17239040, PMID: 33291582 PMC7729997

[21] NIOSH. Hazard Review: health effects of occupational exposure to respirable crystalline silica. National Institute for Occupational Safety and Health (NIOSH). (2002).

[22] Lacasse Y Martin S Simard S Desmeules M. Meta-analysis of Silicosis and lung Cancer. Scand J Work Environ Health. (2005) 31:450–8. doi: 10.5271/sjweh.949, PMID: 16425586

[23] EPA. Supplement to RAGS Part A: Community Involvement in Superfund Risk Assessments. Washington, DC. (1999) 76:30–7.

[24] Dagle GE Wehner AP Clark ML Buschbom RL. Chronic inhalation exposure of rats to quartz In: Goldsmith DR Winn DM Shy CM, editors. Silica, Silicosis and Cancer. Controversy in occupational medicine. New York: Praeger (1986). 255–66.

[25] Johnson NF Smith DM Sebring R Holland LM. Silica-induced alveolar cell tumors in rats. Am J Ind Med. (1987) 11:93–107. doi: 10.1002/ajim.4700110110, PMID: 3028139

[26] Muhle H Kittel B Ernst H. Neoplastic lung lesions in rat after chronic exposure to crystalline silica. Scand J Work Environ Health. (1995) 21:227–9. 8929684

[27] Spiethoff A Wesch H Wegener K Klimisch H-J. The effects of Thorotrast and quartz on the induction of lung tumors in rats. Health Phys. (1992) 63:101–10. doi: 10.1097/00004032-199207000-00011 1325960

[28] Wilson T Scheuchenzuber WJ Eskew ML Zarkower A. Comparative pathological aspects of chronic olivine and silica inhalation in mice. Environ Res. (1986) 39:331–44. doi: 10.1016/S0013-9351(86)80059-7 3007105

[29] Kurihara N Wada O. Silicosis and smoking strongly increase lung cancer risk in silica-exposed workers. Ind Health. (2004) 42:303–14. doi: 10.2486/indhealth.42.303, PMID: 15295901

[30] Steenland K Ward E. Silica: a lung carcinogen. CA Cancer J Clin. (2014) 64:63–9. doi: 10.3322/caac.21214, PMID: 24327355

[31] Radnoff DL Todor MS Beach J. Occupational exposure to crystalline silica at Alberta work sites. J Occup Environ Hyg. (2014) 11:557–70. doi: 10.1080/15459624.2014.887205, PMID: 24479465

[32] Gamble JF. Crystalline silica and lung Cancer: a critical review of the occupational epidemiology literature of exposure-response studies testing this hypothesis. Crit Rev Toxicol. (2011) 41:404–65. doi: 10.3109/10408444.2010.541223, PMID: 21548755

[33] De KNH Musk AW. Silica, compensated silicosis, and lung cancer in Western Australian goldminers. Occup Environ Med. (1998) 55:243–8. doi: 10.1136/oem.55.4.243, PMID: 9624278 PMC1757568

[34] Mcdonald C. Silica, Silicosis, and lung Cancer: an epidemiological update. Appl Occup Environ Hyg. (1995) 10:1056–63. doi: 10.1080/1047322X.1995.10389095

[35] Mundt KA Thompson WJ Dhawan G Checkoway H Boffetta P. Systematic review of the epidemiological evidence of associations between quantified occupational exposure to respirable crystalline silica and the risk of silicosis and lung cancer. Front Public Health. (2025) 13:1554006. doi: 10.3389/fpubh.2025.1554006, PMID: 40093725 PMC11906704

[36] Pelucchi C Pira E Piolatto G Coggiola M Carta P La Vecchia C. Occupational silica exposure and lung Cancer risk: a review of epidemiological studies 1996–2005. Ann Oncol. (2006) 17:1039–50. doi: 10.1093/annonc/mdj125, PMID: 16403810

[37] Soutar C Robertson A Miller BG Searl A Bignon JY. Epidemiological evidence on the carcinogenicity of silica: factors in scientific judgement. Ann Occup Hyg. (2000) 44:3–14. doi: 10.1016/S0003-4878(99)00047-2, PMID: 10689755

[38] Weill H Mcdonald JC. Exposure to crystalline silica and risk of lung cancer: the epidemiological evidence. Thorax. (1996) 51:97–102. doi: 10.1136/thx.51.1.97, PMID: 8658382 PMC472812

[39] Richards JR Brozell TT. Review of previously published ambient air respirable crystalline silica concentration data for use in risk assessment of mineral industry sources. Front Public Health. (2025) 13:2296–565. doi: 10.3389/fpubh.2025.1584841 PMC12075312 40371281

[40] Nishida C Yatera K. The impact of ambient environmental and occupational pollution on respiratory diseases. Int J Environ Res Public Health. (2022) 19:2788. doi: 10.3390/ijerph19052788, PMID: 35270479 PMC8910713

[41] Bhagia LJ. Non-occupational exposure to silica dust. Indian J Occup Environ Med. (2012) 16:95–100. doi: 10.4103/0019-5278.111744, PMID: 23776316 PMC3683189

[42] Gift JS Faust RA. Noncancer inhalation toxicology of crystalline silica: exposure-response assessment. J Expo Anal Environ Epidemiol. (1997) 7:345–58. PMID: 9246596

[43] Bridge I. Crystalline silica: a review of the dose response relationship and environmental risk. Air Qual Climate Change. (2009) 43:17.

[44] Meldrum M Howden PJ. Crystalline silica: variability in Fibrogenic potency. Ann Occup Hyg. (2002) 46:27–30.

[45] Senkayi AL Dixon JB Hossner LR Yerima BP Wilding LP. Replacement of quartz by Opaline silica during weathering of petrified wood. Clay Clay Miner. (1985) 33:525–31. doi: 10.1346/CCMN.1985.0330607

[46] EPA (1999) Risk assessment guidance for superfund (RAGS).

[47] Attfield MD Costello J. Quantitative exposure-response for silica dust and lung Cancer in Vermont granite workers. Am J Ind Med. (2004) 45:129–38. doi: 10.1002/ajim.10348, PMID: 14748044

[48] Cassidy A Mannetje AT Van Tongeren M Field JK Zaridze D Szeszenia-Dabrowska N. Occupational exposure to crystalline silica and risk of lung cancer: a multicenter case-control study in Europe. Epidemiology. (2007) 18:36–43. doi: 10.1097/01.ede.0000248515.28903.3c 17149143

[49] Checkoway H Hughes JM Weill H Seixas NS Demers PA. Crystalline silica exposure, radiological Silicosis, and lung Cancer mortality in diatomaceous earth industry workers. Thorax. (1999) 54:56–9. doi: 10.1136/thx.54.1.56, PMID: 10343633 PMC1745344

[50] Checkoway H Franzblau A. Is silicosis required for silica-associated lung cancer? Am J Ind Med. (2000) 37:252–9. doi: 10.1002/(sici)1097-0274(200003)37:3<252::aid-ajim2>3.0.co;2 10642414

[51] Checkoway H Heyer NJ Demers PA Gibbs GW. Reanalysis of mortality from lung cancer among diatomaceous earth industry workers, with consideration of potential confounding by asbestos exposure. Occup Environ Med. (1996) 53:645–7. doi: 10.1136/oem.53.9.645 8882123 PMC1128562

[52] Ge C Peters S Olsson A Portengen L Schüz J Almansa J. Respirable crystalline silica exposure, smoking, and lung Cancer subtype risks. A pooled analysis of case-control studies. Am J Respir Crit Care Med. (2020) 202:412–21. doi: 10.1164/rccm.201910-1926OC, PMID: 32330394 PMC7465090

[53] Poinen-Rughooputh S Rughooputh MS Guo Y Rong Y Chen W. Occupational exposure to silica dust and risk of lung Cancer: an updated Meta-analysis of epidemiological studies. BMC Public Health. (2016) 16:1137. doi: 10.1186/s12889-016-3791-5, PMID: 27814719 PMC5095988

[54] Rice FL Park R Stayner L Smith R Gilbert S Checkoway H. Crystalline silica exposure and lung Cancer mortality in diatomaceous earth industry workers: a quantitative risk assessment. Occup Environ Med. (2001) 58:38–45. doi: 10.1136/oem.58.1.38, PMID: 11119633 PMC1740036

[55] Shahbazi F Morsali M Poorolajal J. The effect of silica exposure on the risk of lung cancer: a dose-response meta-analysis. Cancer Epidemiol. (2021) 75:102024. doi: 10.1016/j.canep.2021.102024, PMID: 34560363

[56] Sogl M Taeger D Pallapies D Brüning T Dufey F Schnelzer M. Quantitative relationship between silica exposure and lung Cancer mortality in German uranium miners, 1946–2003. Br J Cancer. (2012) 107:1188–94. doi: 10.1038/bjc.2012.374, PMID: 22929885 PMC3461166

[57] Steenland K. One agent, many diseases: exposure-response data and comparative risks of different outcomes following silica exposure. Am J Ind Med. (2005) 48:16–23. doi: 10.1002/ajim.20181, PMID: 15940719

[58] Steenland K Sanderson W. Lung Cancer among industrial sand workers exposed to crystalline silica. Am J Epidemiol. (2001) 153:695–703. doi: 10.1093/aje/153.7.695, PMID: 11282798

[59] Zhou Y Zhang W Wu D Fan Y. The effect of silica exposure on the risk of lung cancer: a meta-analysis. J Biochem Mol Toxicol. (2023) 37:e23287. doi: 10.1002/jbt.23287, PMID: 36642978

[60] Finkelstein MM. Silica, silicosis, and lung cancer: a risk assessment. Am J Ind Med. (2000) 38:8–18. doi: 10.1002/1097-0274(200007)38:1<8::AID-AJIM2>3.0.CO;2-#, PMID: 10861762

[61] Kachuri L Villeneuve PJ Parent MÉ Johnson KC Canadian Cancer Registries Epidemiology Group, and Harris, S. A. Occupational exposure to crystalline silica and the risk of lung cancer in Canadian men. Int J Cancer. (2014) 135:138–48. doi: 10.1002/ijc.28629, PMID: 24272527

[62] Liu Y Steenland K Rong Y Hnizdo E Huang X Zhang H. Exposure-response analysis and risk assessment for lung Cancer in relationship to silica exposure: a 44-year cohort study of 34,018 workers. Am J Epidemiol. (2013) 178:1424–33. doi: 10.1093/aje/kwt139, PMID: 24043436 PMC4522915

[63] Thompson D Qi C. Characterization of the emissions and crystalline silica content of airborne dust generated from grinding natural and engineered stones. Ann Work Exp Health. (2023) 67:266–80. doi: 10.1093/annweh/wxac070, PMID: 36219621 PMC9928769

[64] Checkoway H Heyer NJ Seixas NS Welp EAE Demers PA Hughes JM. Dose-response associations of silica with nonmalignant respiratory disease and lung Cancer mortality in the diatomaceous earth industry. Am J Epidemiol. (1997) 145:680–8. doi: 10.1093/aje/145.8.680, PMID: 9125994

[65] Birk T Mundt KA Crawford L Driesel P. Results of 15 years of extended follow-up of the German porcelain workers cohort study: lung cancer and silicosis. Front Public Health. (2025) 13:1552687. doi: 10.3389/fpubh.2025.1552687, PMID: 40171434 PMC11959091

[66] Almberg KS Halldin CN Friedman LS Go LHT Rose CS Hall NB. Increased odds of mortality from non-malignant respiratory disease and lung cancer are highest among US coal miners born after 1939. Occup Environ Med. (2023) 80:121–8. doi: 10.1136/oemed-2022-108539, PMID: 36635098 PMC10428099

[67] SEER (2024) Cancer of the lung and bronchus—Cancer stat facts. Available online at: https://seer.cancer.gov/statfacts/html/lungb.html (Accessed January 8, 2025).

[68] Villeneuve PJ Mao Y. Lifetime probability of developing lung cancer, by smoking status Canada. Can J Public Health. (1994) 85:385–8. PMID: 7895211

[69] Marrocco A Dy ABC Ortiz LA Slavin TJ Dotson S Stevens M. Silica and Silica Compounds. In Patty’s Toxicology. (eds Paustenbach D. J. Farland W. Greim H. Klaunig J. Levy L.). (2025). doi: 10.1002/0471125474.tox011.pub3

[70] Pairon JC Brochard P Jaurand M Bignon JY. Silica and lung cancer: a controversial issue. Eur Respir J. (1991) 4:730–44. doi: 10.1183/09031936.93.04060730, PMID: 1653712

[71] Baum L Arnold TC. Silicosis. Treasure Island, FL: StatPearls Publishing (2023). 37603636

[72] Fubini B. Surface chemistry and quartz Hazard. Ann Occup Hyg. (1998) 42:521–30. doi: 10.1016/S0003-4878(98)00066-0, PMID: 9838865

[73] Lapp NL Castranova V. How silicosis and coal workers’ pneumoconiosis develop – a cellular assessment. Occup Med. (1993) 8:35–56. 8384379

[74] Johnston CJ Driscoll KE Finkelstein JN Baggs R O’Reilly MA Carter J. Pulmonary chemokine and mutagenic responses in rats after subchronic inhalation of amorphous and crystalline silica. Toxicol Sci. (2000) 56:405–13. doi: 10.1093/toxsci/56.2.405, PMID: 10911000

[75] OEHHA. California Office of Environmental Health Hazard Assessment (OEHHA) February 2005. Final report: Silica (crystalline, respirable) chronic toxicity summary. State of California: California Environmental Protection Agency (2005).

[76] Haber LT Bradley MA Buerger AN Behrsing H Burla S Clapp PW. New approach methodologies (NAMs) for the in vitro assessment of cleaning products for respiratory irritation: workshop report. Front Toxicol. (2024) 6:1431790. doi: 10.3389/ftox.2024.1431790, PMID: 39439531 PMC11493779

[77] Vallyathan V Kang JH Van Dyke K Dalal NS Castranova V. Response of alveolar macrophages to in vitro exposure to freshly fractured versus aged silica dust: the ability of Prosil 28, an organosilane material, to coat silica and reduce its biological reactivity. J Toxicol Environ Health. (1991) 33:303–15. doi: 10.1080/15287399109531529, PMID: 1649918

[78] Vallyathan V Castranova V Pack D Leonard S Shumaker J Hubbs AF. Freshly fractured quartz inhalation leads to enhanced lung injury and inflammation. Potential role of free radicals. Am J Respir Crit Care Med. (1995) 152:1003–9. doi: 10.1164/ajrccm.152.3.7663775, PMID: 7663775

[79] Jaganathan H Godin B. Biocompatibility assessment of Si-based nano- and micro-particles. Adv Drug Deliv Rev. (2012) 64:1800–19. doi: 10.1016/j.addr.2012.05.008, PMID: 22634160 PMC3465530

[80] Kim IY Joachim E Choi H Kim K. Toxicity of silica nanoparticles depends on size, dose, and cell type. Nanomedicine. (2015) 11:1407–16. doi: 10.1016/j.nano.2015.03.004, PMID: 25819884

[81] Goldsmith DF Ruble RP Klein CO. Comparative cancer potency for silica from extrapolations of human and animal findings. Scand J Work Environ Health. (1995) 21:104–7. 8929704

[82] Englen MD Taylor SM Laegreid WW Silflow RM Leid RW. The effects of different silicas on arachidonic acid metabolism in alveolar macrophages. Exp Lung Res. (1990) 16:691–709. doi: 10.3109/01902149009087889, PMID: 1964411

[83] NTP. (2014). Silica, crystalline (respirable size). Report on carcinogens. Thirteenth edition. Research Triangle Park, NC: National Toxicology Program. Available at: http://ntp.niehs.nih.gov/ntp/roc/content/profiles/silica.pdf (Accessed July 30, 2015)., PMID: 21784044

[84] Shi X Ding M Chen F Wang L Rojanasakul Y Vallyathan V. Reactive oxygen species and molecular mechanism of silica-induced lung injury. J Environ Pathol Toxicol Oncol. 20 Suppl. (2001) 1:85–93., PMID: 11570677

[85] R Studio Team. (2020). RStudio: Integrated Development for R. RStudio, PBC, Boston.

[86] Hack CE Haber LT Maier A Shulte P Fowler B Lotz WG. A Bayesian network model for biomarker-based dose response. Risk analysis: an official publication of the Society for Risk Analysis. (2010) 30:1037–1051. doi: 10.1111/j.1539-6924.2010.01413.x, PMID: 20412521

[87] Muhle H Takenaka S Mohr U Dasenbrock C Mermelstein R. Lung tumor induction upon long-term low-level inhalation of crystalline silica. Am J Ind Med. (1989) 15:343–346., PMID: 2539015 10.1002/ajim.4700150309

[88] Muhle H Bellmann B Creutzenberg O Dasenbrock C Ernst H Kilpper R. Pulmonary response to toner upon chronic inhalation exposure in rats. Fundam Appl Toxicol. (1991) 17:280–299. doi: 10.1016/0272-0590(91)90219-T 1662648

[89] Holland L, Gonzales M, Wilson J L Gonzales M Wilson J. Pulmonary effects of shale dusts in experimental animals. In: Health Issues Related to Metal and Nonmetallic Mining. Wagner W, Rom W, Merchand J, editors. Boston MA:Butterworths, (1983) 485–496. doi: 10.3155/1047-3289.59.11.1287, PMID: 19947110

[90] Holland L Wilson JMI Tillery M. Lung cancer in rats exposed to fibrogenic dusts. In: Silica, Silicosis, and Cancer: Controversy in Occupational Medicine. Goldsmith DR, Winn DM, Shy CM, editors. New York: Praeger, (1986) 267–279., PMID: 17179757

[91] EPA. Ambient levels and noncancer health effects of inhaled crystalline and amorphous silica: Health issue assessment. Research Triangle Park, NC: U.S. Environmental Protection Agency. EPA600R95115. (1996) Available at: http://ofmpub.epa.gov/eims/eimscomm.getfile?p_download_id=4608 (Accessed October 6, 2015).

